# Adolescent Health Services in a Primary Care Facility in Northern Ghana: A Qualitative Investigation of Patients’, Guardians’, and Staff’s Perceptions of Care

**DOI:** 10.21203/rs.3.rs-7465828/v1

**Published:** 2025-09-19

**Authors:** Joshua Akurugu Abaah, Olivia Soliku, Mohammed Hafiz Kanamu, Scott R. Ikeda, Abubashr Adamu Mohammed, Stephen Tetteh Engmann, David Sesime Ahadzi, Marie A. Brault, Alhassan Abdul-Mumin

**Affiliations:** Tamale Teaching Hospital; Tamale Teaching Hospital; Tamale Teaching Hospital; Montefiore Medical Center; Tamale Teaching Hospital; University of Cape Coast; Tamale Teaching Hospital; NYU Grossman School of Medicine; Tamale Teaching Hospital

**Keywords:** Adolescent health services, Primary care, Perception of care

## Abstract

**BACKGROUND:**

Ghana’s population of 30.8 million people is predominantly young, with a median and mean age of 21 and 25.2 years, respectively. Yet, there are limited studies on quality of care for Adolescent and Young Adult (AYA) aged 10–24years in Ghana. To better understand barriers and facilitators to AYA primary care, we conducted a qualitative study at the Tamale Teaching Hospital polyclinic as part of a broader quality improvement initiative for youth care.

**METHODS:**

Convenience sampling was used to identify and recruit 12 youth, 8 guardians, and 22 staff for in-depth interviews and focus group discussions. In-depth interviews were conducted with AYAs and guardians, and focus group discussions were conducted with staff. Interviews and focus group discussions explored topics including: previous care experiences and reasons for visits, experiences and perceptions of service at registration or booking points, nursing services, consultations with doctors, services at the laboratory, pharmacy, and cash payment point. The study employed inductive and deductive reflexive thematic analysis of interviews and focus group discussions. All qualitative data were transcribed. The qualitative software NVivo 14 was used for coding and theme development.

**RESULTS:**

Five themes emerged: Healthcare quality and accessibility, systemic and administrative challenges, Autonomy, confidentiality and privacy, adolescent health conditions and needs, and participants’ perspectives on improvement. The major barriers to adolescent responsive care are system inefficiencies and negative staff attitudes, and the major facilitators are trust and confidence in doctors’ competence, perceived superior care in a Teaching Hospital, and convenient services and proximity.

**CONCLUSION:**

AYAs and guardians desire convenient, easily accessible, and high-quality care, delivered by a friendly and trusted team. The staff members envision the same kind of care for youth and believe that restructuring mainstream care and providing adequate training makes this possible.

## BACKGROUND

According to the World Health Organization, adolescents are least well served by existing health services(1). Although globally, efforts towards quality adolescent healthcare have been made, the progress is slow. Out of the 109 countries reviewed by WHO, three-quarters of existing services only focused on sexual and reproductive health. The lack of comprehensive and integrated approaches also makes these existing efforts weak(1). The situation in Ghana is similar(2). Agblevor et al. also found that only 4 (17%) out of the 23 stated strategies and programs in the National Adolescent Health Service Policy and Strategy were fully implemented, with limited integration of youth programming into general mainstream services of the Ghanaian healthcare system(3).

Many factors affect the dynamics of adolescent healthcare. Adolescence is a period of rapid biological and psychosocial growth with behavioural patterns that make the health risks and implications of this group unique. Adolescents may look mature physically, yet their cognitive control networks for relational reasoning are not mature. So they explore, experiment and take risks in affective social context, often with poor planning, judgment, and control(1, 4–10). At times, peer influence from adolescents with antisocial behaviours worsens the risk profile. Healthcare workers must have a good understanding of these unique characteristics of adolescence and the inherent behavioural patterns and health implications to enable them serve them well. Therefore, a responsive healthcare system ought to have healthcare workers who are knowledgeable, non-judgmental, considerate, easy to approach, and trustworthy. The services should also be affordable, convenient, accessible, and appropriate to meet all their needs(11)

Parents, guardians, and the community also act as gatekeepers in adolescents’ lives. Societal norms prescribe what is acceptable and often frown at premarital sex, and contraceptive use is often forbidden. Such societal norms can contribute to unintended pregnancies and unsafe abortions among adolescents. Community perceptions formed from these norms, and home environment, power structure, and rules all heavily influence adolescent healthcare choices, health literacy, acceptance, patronage, or rejection of health services(1, 2, 12–19).

Healthcare system and facility-specific characteristics can either enhance or hinder quality healthcare for adolescents and young adults. Adolescents generally prefer services that are convenient, affordable, and accessible. They also want it to be offered by a competent adolescent-friendly healthcare provider(20). Traditionally, healthcare system infrastructure and training are often tailored towards pediatric and adult patients. The care may be accessible but hostile and not affordable or not convenient because it does not support adolescent comfort, autonomy, privacy, and confidentiality(17, 21). There is also a healthcare provider knowledge gap, and again a lack of or ineffective health-protecting and promotive policies and laws for adolescents.

Also, health literacy is key to adolescent empowerment. The 2016–2020 Ghanaian Adolescent Health Service Policy and Strategy situation analysis of adolescent healthcare indicated that adolescents in Ghana have poor access to appropriate health information, and their utilization of health services is also poor. As already stated, the implementation of the policy guidelines remains a big challenge, and adolescent health literacy remains low(3, 17, 21–23). The aim of the study was to understand staff,’ Guardians’, and AYAs’ perception of the clinical services offered to Adolescents at Tamale Teaching Hospital Polyclinic to enable us to ascertain the perceived facility-specific characteristics and staff-related issues that enhance or hinder quality healthcare delivery to adolescents and young adults, and how to improve upon or establish needed services.

## METHODS

### Study Design

This study employed a qualitative research design, utilizing in-depth interviews and focus group discussions to gain insights into the experiences, perceptions, and opinions of AYAs, guardians, and healthcare staff and their recommendations regarding adolescent health services at a primary care facility in Northern Ghana. The interview guide used for the study was designed by the authors. It is submitted separately as a supplementary file.

### Study Setting

The study was conducted at the Tamale Teaching Hospital Polyclinic, a primary care facility located in northern Ghana. This facility serves as the entry point for various healthcare services and the primary care division for the Teaching Hospital within the region. Adolescents within the Tamale Metropolis, the surrounding districts in the Northern Region, and its neighboring districts and regions, as well as students attending schools in the Northern Region, access its services. It has a 32-bed capacity for inpatient care. The study also focused on understanding the facility’s strengths and challenges in meeting the unique needs of adolescent patients.

### Sample Size

The study recruited a total of 42 participants, comprising 12 adolescent and young adult patients, 8 guardians, and 22 healthcare staff members. The stated numbers of participants in each data set achieved depth, richness, and diversity.

### Participants

The recruited AYAs and their guardians were patients who had visited the Polyclinic at least once in the previous year for healthcare reasons. Three inpatients and one outpatient declined to participate. Of the AYAs recruited, the age range was from 14 years to 24 years. Age group 10–13 years were excluded because those between the ages of 10–12 years are seen at the Pediatrics and Child Health Department, and an attempt to interview a 13-year-old who assented and parent consented did not prove fruitful. 4 received both inpatient and outpatient services, and 8 received only outpatient services. The staff were those who had worked there for at least one year. Three staff members declined to participate, citing that they were not interested.

Four focus groups were organized for the different cadres of staff. The nurses’ focus group consisted of 7 members, and the prescribers’ group comprised 6 members, including two Family Medicine Residents, two Medical officers, and two Physician Assistants. There were four managers: a nurse manager, an administrator, a Pharmacy manager, and an Accountant. The other staff focus group consisted of two laboratory staff, two health information staff, and one pharmacy staff member. The least years of work at the Polyclinic was two years, and the most was nine years.

### Procedure

The research team consisted of four doctors, of which two were staff members of the Polyclinic and the other member was an administrator of the Polyclinic. The Principal Investigator had training in qualitative research, and the study team included two experts in this field. There were prior encounters with two inpatient participants interviewed. However, efforts were made to minimize social desirability bias, and where possible, we avoided recruiting and interviewing those with prior encounters with research staff. Convenience sampling of patients and guardians who had at least one visit to the Polyclinic in the past 12 months, and staff who had worked at the facility for at least one year, was done. The interview guide was designed, reviewed, pre-tested, and revised by three people; one of whom has very good expertise in qualitative research. This baseline study aimed at understanding the factors that facilitate or hinder service delivery as well as the facility’s ability to deliver quality healthcare, both at OPD and inpatient, to AYAs. We also sought to understand the perception of healthcare providers on how the Polyclinic is doing in relation to service delivery to this group. So at least two staff members participated from all the service points of the Polyclinic. The participants were given the patient information leaflet to read, and the research staff provided an explanation in English to facilitate a clearer understanding. Demographics of those who consented were first taken, and then they were interviewed using an open-ended interview guide. In-depth interviews were done for all 12 AYAs and 8 guardians to gain full insight into the rich and diverse lived experiences, perceptions, and suggestions. The focus groups were intended to ascertain the collective views and opinions of the designated staff groups, rather than individual experiences. The questions were structured in such a way as to ensure that responses covered the disease conditions they presented with, and if there were any unaddressed health problems that required healthcare provider attention. They also sought to cover all service points and all cadres of workers, and the physical layout of the place and organization of work. All participants were given the opportunity to contribute to any needed changes and to suggest the best way to plan and deliver healthcare services for AYAs. The interviews lasted a minimum of 30 minutes and a maximum of one (1) hour and were recorded using voice recorder and transcribed and themes generated using NVivo 14. The review of the manuscript was first done by three experienced researchers among the team and finally by one with long standing experience in qualitative research.

### Analysis

The concept and design of the study prior to analysis were not based on any theoretical or conceptual framework. The data sets for AYAs, Guardians, and staff were coded differently. The codes and themes generated from these data sets became the master data set. Theme commonalities were generated for this analytic direction. Reflexive thematic analysis, conducted by three analysts to reinforce rigor and quality, was performed using the six steps outlined by Braun and Clark(24–28). The inductive and deductive analysis of the master data set resulted in five theme commonalities; Healthcare quality and accessibility, Systemic and Administrative Challenges, Autonomy, Confidentiality and Privacy, Adolescent Health Conditions and Needs, and Participants’ perspectives on improvement. The write-up employed both inductive and deductive meaningful generation of knowledge from the data sets and certain conceptual frameworks relevant to this study. The conceptual frameworks were integrated in a flexible and iterative manner during familiarisation, coding, and theme development. Understanding of the adolescent period and their unique behavior, perceptions, and decision-making patterns made behavioral science models very necessary. Verbal models such as social motivation, reward sensitization, and distraction were crucial in this regard. An increase in social motivation from high social value, validation, and acceptance is notably common among adolescents, and social influence from peers creates a dramatic effect on adolescent risk-taking, and the risky behaviour is often associated with altered representation of the reward, with poor self-control and diminished cognitive skills due to the immature cognitive control systems of the brain(6, 7, 9, 29).

The socioecological framework was needed for deductive analysis by aiding in the understanding of the gatekeeper effect of guardians and the community on their healthcare decision-making and interactions(18, 19, 30). Also gatekeepers must seriously be considered in healthcare planning and implementation.

For the staff, we viewed the opportunities that exist for restructuring of care with the incorporation of the AYAs’ and guardians’ recommendations with the self-determination theory lens(31, 32). Deductively, we assumed that if there is inherent interest in becoming more competent and becoming an adolescent-friendly staff, as well as working innovatively to achieve this, then strategies that aim at satisfying these basic psychological needs from a self-determination theory perspective, such as autonomy support, relatedness support, and competence support, will greatly improve care.

## RESULTS

A total of 42 participants, with a male to female ratio of 1:1, consisting of 12 adolescent and young adult patients, 8 guardians, and 22 healthcare staff participated in the study. 91.7% of the AYAs were students with 4 in a University, 1 in a Nursing and Midwifery College, 1 in a Junior high, and 6 in a Senior High school. The oldest among them (24 years) had completed a Technical University and was working. The median and mean age were 19.5 and 19.3 years respectively.

The parents/guardians were eight(8); 4 were biological mothers, 2 were biological fathers, 1 was an uncle, and 1 was the Father’s friend who came with the father because of a language barrier. 4 were University graduates, 2 attained Junior high,, 1 Senior High, and 1 form 4. The mean age among the guardians and staff was 48 years and 33.7years respectively.

Table 1 shows the Demographic data of participants.

## HEALTHCARE QUALITY AND ACCESSIBILITY

The quality of relationship and interactions in AYA healthcare was weighted higher and more valuable to adolescents and their guardians compared to staff-perceived privacy and confidentiality needs. Any disconnection or dismissive, unconcerned, rude, or inattentive behavior was seen as a serious barrier to quality healthcare delivery. Respect for patient dignity, values, and preferences, which are globally acclaimed as best strategies and underpin Patient Centered Care and Adolescent responsive service policy, was clearly demonstrated by this study as a key pillar of quality healthcare(11, 33–36)

“Some of the nurses, although you have not yet spoken to them, but how they appear scares you a bit. So like sometimes you come and you see a nurse and how she is moving, even her posture and her body language makes you afraid to even approach her to ask her something. Yeah, some of them if they are talking, they don’t speak calmly, like they are shouting on you and they are just like authoritative. They are just doing, so it makes you feel scared to even approach them or ask them a question. And even if you want to, if you ask them a question and they are going to reply, you yourself, you will not be happy, like they’re shouting on you.” AYA 7

“The nurses I come in contact a lot, and it’s like most of them. yes, it’s like sometimes when you’re even talking to them, they don’t care, yes, because they don’t put themselves in your shoes. They just act; so some of them are actually very disrespectful.” AYA 3

It was also evident across the staff focus groups that some staff members’ attitudes towards AYAs were repulsive, rude, and created fear among AYAs and did not respect their dignity. The negative attitude or behaviour, which creates fear among AYAs as reported above, was a key indicator of poor performance in delivering healthcare to AYAs, especially among managers, prescribers, and other staff focus groups.

“Very poor. Very, very poor. Yeah, because we see them as children. So we treat them like children. In some of the consulting rooms, you go and the doctors are shouting at them. They come to the pharmacy, we talk anyhow to them because we think they are children, you see the way we treat adults, that’s not the same way, but they need special attention as compared to the adults, because they are more emotional,” Manager 3

“To me I think, I score it not very good. The way they have been handled by the nurses at the OPD because of their age; they say something and the nurse will descend on them. Sometimes one has to intervene between the two of them” Prescriber 5

Some of the Prescribers argued that, due to inadequate training, some healthcare workers don’t understand adolescents well and treat them in a dismissive manner. It was evident from the examples given that certain unusual behaviors in adolescents, which require tact, skill, and knowledge to handle, often attract unfair treatment from those who are not skilled enough to address these issues.

“Sometimes I think because they don’t know how to handle some of these things and because of the way they act, they are not handled well but is it not all that bad” Prescriber2

It was, however, a surprising finding from both AYAs and their guardians that prescribers were perceived as being nice, approachable, friendly, calm, and easy to relate to and open up to.

“Yes, the doctors are friendly, and even the last time I came the doctor I met, she was very friendly and I’m always happy if I meet friendly people I can talk.” AYA 7,

“Like when they are talking, they are calm, they don’t shout on the patient.” AYA 4. “Yeah, the one who was in charge, she really treated her well, the psychology she used, even the way she approached us and the child, she was very calm. My wife was always happy with her, even the one who received us in the night he was also very good” Guardian 8.

“This place if you come the doctors have time with patients especially the younger ones. They take good care of us.” Guardian 4.

They also had confidence in prescribers’ ability to diagnose and handle their healthcare needs, reporting good outcomes. This further fostered their initial confidence and trust, rewarding their decision based on their perceived better care in the Facility.

“I have believe with this hospital. If you brought a patient here no matter how it is they will take care of him and he will be healed.” Guardian 3

One other feature of healthcare quality was patient prioritization. AYAs and their guardians value fairness in healthcare. They expect a structured, transparent system that prioritizes patients based on severity of illness rather than arbitrary decisions. The observed ineffective triage system to help prioritize patient care was a worry to them.

“There should be fairness in the healthcare delivery, yes, like people who come in with emergency situations. Yes, because we were waiting to take our lab reports, and there was a girl lying there. You could see that she was shivering. But then she had to wait outside until the results were ready. So in this case I don’t know. It’s, not nice. Yeah. So I think there should be fairness.” AYA 3

“There can be a situation whereby a patient may be in pain and will need serious intervention, but because their labs are delayed, the patient can’t be attended to. Which can cause maybe untimely death to some patients” AYA 5

“The girl was having temperature! When they got to the lab, they said they have to pay for the lab and they were not having money yet. So they were lying down, calling, to see if they will get money. The girl, I felt it.. She was lying at the veranda there spiking temperature, the labs were not done because she can’t pay for the labs.” Guardian 2

“And when you come here and there are plenty people and your sickness is high, you want to see them so that they will take care of you so that you will see the doctor. They will leave you to lie down and suffer. I am talking about the nurses because if you come here it is their responsibility to take care of you first. So if you come here and they leave you and something happen to you I will say it is their fault” Guardian 4

Accessibility is a key tenet of adolescent-responsive services (11). AYAs don’t only look for healthcare services that are accessible, but they must also be convenient. Convenience has different forms to them and include comfort; *“In the polyclinic, the doctors here they’re very free”AYA 6*, ease of access to a doctor, payment relief and modalities, and easy navigation, which to them are as important as accessibility.

“I see to it that this place is better, and this place is easy to meet the doctor than when you go to other places. And there are many doctors here. So when you are here it’s very easy to get to a doctor. When you come to this place, people are there to just direct you where to go.” AYA 4

“My hostel isn’t far from here, so I think it would be easier.” AYA 1

“For convenience, because my mom works here and she knows a lot of people here, so it’s much easier than going to a different facility where I don’t know anybody. So coming here makes it faster for me.” AYA 3

## SYSTEMIC AND ADMINISTRATIVE CHALLENGES

The Adolescent and Young Adults and their guardians have the notion that hospitals that they perceive as “superior” in healthcare should have structures and systems in place for effective and efficient care. They expect not just a nice edifice with modern equipment but also healthcare workers who deliver care with high level of professionalism.

“This is the major hospital in the region, and of course you should be the best. In terms of delivery, it should be very good, since it’s the face of the northern region, the face of the hospitals, not in the northern region alone, but in the whole northern sector of Ghana. Yes. And the doctors and the nurses should also be friendly. They should be easy to approach.” AYA 7

This expectation was not met as they saw that such structures and systems were non-existent at the Polyclinic which is the face and entry point to the Tamale Teaching Hospital. The repeated network connectivity issues for the electronic health record system, lack of constant engagement with patients to explain to them what was happening during delays, no communication about the laboratory turnaround times and ineffective supervision of doctors to make them accountable to curb doctors’ lateness to and/or absence from work were seen as administrative flaws of the Polyclinic.

“Due to the network issue, the patients were there, no one really came out to speak to them about what was happening, to explain to them why they were waiting so long and why they were not being attended to” AYA 3

“Sometimes when the LHIMS is down like that you see that patients will just be suffering and there is no education. You know the patient does not understand what is LHIMs, I will feel you are not doing your work, meanwhile the machine is not allowing you to do your work. But I will not understand if I am not educated.” Guardian 2

“Yes, the last time I came here, it was really rough that day, that day when I came it was crowded and I waited. It got to my turn and the light went off. And when the lights came it went back off again, and the doctor left, and another doctor came. So we sat there for almost getting to one hour thirty minutes and another doctor came. When that doctor came, we gave that doctor space to prepare, and we were entering, when the first person entered, they said network issues. So that day it was very, very bad.” AYA 7

“And I don’t know if maybe sometimes too, maybe you break to have your lunch or super, like you should always let the people know that. Oh, we are going here to do this and come. But you shouldn’t just leave the patients sitting like that unaware. Then sometimes we’ll be talking to you then you won’t mind us and you’ll be like oh we are disturbing you or something. But if we are not feeling well like we won’t come here.” AYA 8

Almost all the staff saw network connectivity problems as a major hindrance to effective care, and that of the lack of proactive measures to address it or help in patient consultation; enshrined in administrative inertia and lack of commitment. Administratively they also expected a better organization of the care to provide adequate privacy and confidentiality.

“The LHIMS, if there is no network it is a major challenge because this week, I learn mostly in the mornings the network is not working, even if you are done with the labs you can’t send the results” Lab staff 2

“The triage is too open because when they come you need to ask questions. So because it’s open if he or she is sitting down and speaks, other patients will hear. So I think because it is very open, when they come they might not even tell you their needs or their problems.” Nurse 1

“Even the way our consulting rooms are. You sit here having conversations, the person in the next consulting room hears whatever is going on. So it does not give adequate security to voice out whatever is worrying them.” Prescriber 5

## AUTONOMY, CONFIDENTIALITY AND PRIVACY.

Parents, guardians and community members are gatekeepers and play critical role in acceptance of services offered to AYAs, even in adolescent and Youth friendly facilities. Evidence suggest that lack of confidentiality, privacy and trust is a major barrier to adolescent healthcare (1, 16, 22, 37, 38). The guardians and staff in this study acknowledged the critical impact of these principles on quality of care. And it was heartwarming to note that almost all guardians, except two, endorsed the need for adolescent autonomy, privacy and confidentially in healthcare. One guardian related to it this way.

“That’s the best. It is the child who is sick, you the father you don’t know what is in the body, if only the child can talk for himself, you will just sit aside and the child will talk whatever is worrying him and then if money needs to be spent that is your duty but to talk about the sickness you the father you are out of it.” Guardian 3.

“As they are like that they have things in them they can’t share it, so when they come to the Polyclinic, the nurses’ station where they take the vitals, the nurses should educate them, feel free to tell the doctor all your problems. The doctor is there for you, don’t fear the doctor, tell the doctor your problems, whatever is disturbing you, he is the one to help you, when that is given to them they enter with vim.” Guardian 2

“You know sometimes, the children are afraid of their mothers. As for me I don’t have problem with it but I don’t know about other parents” Guardian 4

One of the two, initially said; *“that’s your father or your mother. Why don’t you want him or her to also take part or hear? What secret do you have to prevent him not to hear? People have been doing that, but I don’t agree to that, no!” Guardian 8. Y*et paraphrasing to help him understand the value of it as required in WHO global quality standards(37), he rescinded his initial position and said; “*OK, what you have said now I understand you, especially if she is a girl, a grown up girl, there are certain things she might not like her father or mother to hear. But somebody else, especially if a doctor is asking; only as you’ve been doing, that’s your work, you have been trained for that. I heard you say it is confidential. So I’ll just advise that it is not bad you still keep it on*.” Guardian 8.

What however was not explored was the type or package of health service that this endorsement is meant for. Some of the staff also related how it is impacting care. As already mentioned under the systemic challenges about the lack of privacy and possibly trust at the triage area, a female adolescent who had condom retained after sexual intercourse refused to open up to the triage nurses.

“Recently, a 17 year old girl came; when she came, we were all ladies sitting at the nurses table…, We asked her why she wanted to see a doctor, she just said she wasn’t feeling well. So we tried to inquire what she was feeling, so she just said she wasn’t feeling well. She waited in the queue till she got into the doctor’s room…., then the doctor now called us that why didn’t we inquire from her what was wrong with her then she was now coming in late? So we told the doctor that we asked her twice and she just said that she wasn’t feeling well. She needed to see a doctor. Her problem was she had a condom that was slipped inside her for two days” Nurse 4

The nuance on the general effect of the lack of privacy from all the nurses was almost the same and one of them summed it up this way.

“At the OPD, the issue would have been the way the triage is set up…, but if there was privacy, maybe there was a bar, to prevent people from hearing, they could have opened up.” Nurse 1

At the consulting rooms, doctors are not able to examine Adolescent patients thoroughly due to lack of privacy and others also stated that having many people and guardians in the consulting room hinders their openness.

“I also think that privacy, confidentiality; patient is in the consulting room and there are a lot of people in the consulting room, anyway, it’s a teaching center, and the patient is not able to voice out the main thing. They will just be beating about the bush until someone leaves the consulting room and they now see that it is between patient-doctor and they now voice out their problems.” Prescriber 4.

“This age group, some of them come with their parents. It is good to invite the child first, exclude the parent because some of them have been put under pressure, questions and other things, so when they come and the parent enters the consulting room with them they will never open up.” Prescriber 3.

“So I think that because of the sensitivity of most of the conditions they present, what they actually want is confidentiality.” Prescriber 5.

One of the AYAs was emphatic about the importance of these crucial practice principles. She came with vaginal infection and the issues discussed above played.

“Like today when I came I met two guys at the nurses’ station,…, And then when they started asking me questions even though I gave them some reasons for coming, but due to certain things I wasn’t able to answer them and then they later realized that I was kind of shy. It was opened. Even if you come to health sector like this, If you go inside the consulting room and then you meet a doctor and then there is a second party in, it is going to be difficult for you to say it” AYA 10.

More importantly the Adolescent and Young Adults saw healthcare services that are organized to provide autonomy, confidentiality and privacy as the preferred approach and also welcomed consultations that will explore every aspect of their health. However one of them saw friends and colleagues as trusted persons to discuss confidential matters about her life than doctors.

“Young ladies are underage and mostly wouldn’t want their parents to know certain things about their reproductive health. So. If they are given the chance to talk to their doctor in the absence of their parents, I think it would be great. Yes, it would help, and it should be done.” AYA 12

“I will say that this your initiative is good if you guys are able to implement it and I think it is going to help many young people” AYA 6

## ADOLESCENT HEALTH CONDITIONS AND NEEDS

The Health conditions that made them visit the Tamale Teaching Hospital Polyclinic were curative in nature; and included communicable and non-communicable diseases. The other predominant health needs of AYAs that participants identified was sexual and reproductive health needs. Bullying and substance use were mentioned as the other issues that affect AYAs health. Anticipatory guidance was a much needed service to AYAs according to their guardians.

### Sexual and reproductive

“And most of these things we the ladies when you’re growing up, we are getting changes in your body, like you don’t know who to talk to.” AYA 8.

#### Infection-related:

“The first one was malaria. I was expecting malaria. So I came to the hospital for confirmation.” AYA 3

#### Substance Use:

“The substance abuse, it’s something serious, especially we those handling the substance at the Pharmacy. Most of them don’t come to just talk to you about being addicted to a particular substance, they don’t come to tell you that. They don’t say it, at the end of the day they want to find out about accessibility to that substance. They will ask you that if I pay for this, will you give me?” Pharmacy staff

### Bullying

“Most often they come with the hysteria nature because of bullying by their seniors in school. So they tend to intensify it so that it will look like something big. Because I have encountered several students in that nature, they are been bullied by their seniors.” Prescriber 3

The AYAs and Guardians in this study value anticipatory guidance and the psychosocial history consultation techniques (HEEADSSS) intended to help unearth problems that are not normally talked about in tradition consultation style as a useful tool. We enquired about their opinions on it and they all accepted it as a necessary tool. One guardian didn’t mince words and said; *“I even love it that way” Guardian 1.*

“She has a sickle cell and if she is to marry he has to go for a test. So I see it very necessary for parents.” Guardian 8

“And then the most important thing in life you doctors should advise we parents, if you want your kids to be healthy, unless you give them the food that the kids need. Your side is to advise us how to make them somebody tomorrow”. Guardian 3

It was surprising that none of the AYAs came because of psychosocial issues, yet one was ever addicted to pornography and masturbation and needed help but never knew there was such help in the hospital. This might be due to lack of knowledge of existing services due to low health literacy(23, 29). On the other hand, such consultation opportunities don’t exist in mainstream clinical service delivery so that could have been the other limitation.

## PARTICIPANTS PERSPECTIVES ON IMPROVEMENT

AYAs and their guardians treasure healthcare providers who are friendly, approachable, empathetic, patient and actively listens to the concerns of young people. Healthcare providers with these qualities foster greater trust and openness among AYAs and this was evident in this study between doctors and AYAs. Also the staff’s views were that adolescent-responsive services are sine qua non for quality care for this population. Restructuring mainstream services to improve quality and make it adolescent friendly featured in what should be done to make healthcare more responsive, accessible and appropriate for adolescents.

“I think patient education, yes, so in the sense that, I’m using today as an example; due to the network issue, the patients were there, no one really came out to speak to them. So if there’s one thing I will change that will be It. So instead of just allowing them to sit there and think maybe you don’t care, you can actually explain to them what’s happening……” “I think there should be fairness in the healthcare delivery. Yeah, so I think there should be fairness. And the attitude towards the work is very important.” AYA 3

“Especially the nurses they should be very friendly because they are the people we meet first, if I come here and I’m seriously sick, even the introduction alone can either make me more sick or a bit well. So if I come and I meet you and you frown your face and I’m talking to you and you are shouting at me, I even get more sick, but if I come and you are happy, you try to relate with me well and make me feel comfortable and encourage me with that, I’ll be able to get back on my feet very fast.” AYA 7

“The supervision of the workers should be strong” AYA 8.

Other views were that getting a dedicated clinic and staff as a stand-alone adolescent services/Clinic will greatly improve their care.

“I think we should get a space that is separated from the public eye where they can come confidently to get their services. We should have dedicated, if it’s possible, dedicated physicians or nurses, so that in case young people at that ages come we should be able to take care of them faster” Nurse 3

Others indicated that the curricula for training healthcare workers should include modules that make healthcare workers more capable and skillful to take care of adolescents. And others also called for in-service training.

“At policy level, we should start looking at how they can incorporate that into the training institutions, so that going forward we also understand them before we come out to practice. So that we can do the postgraduate things to deepen the knowledge.” Prescriber 1

“We also have to do some form of in-service training once a while to also educate the staff about how to handle this age group.” Prescriber 2

What was strikingly missing was that, aside curative and anticipatory guidance, AYAs and their guardians were silent on the non-existent services; especially the widely acknowledged reproductive health needs of this populations as mentioned by AYAs themselves, some guardians and most staff. Staff as well did not fully articulate all service packages or components that needed to be added to existing services to make the services comprehensive, holistic and well integrated

## DISCUSSION

The findings in this study agrees with the WHO assertion that adolescents are least served well by existing healthcare systems in many parts of the world(1). Although, there is increasing attention given to Adolescent healthcare with development of National Adolescent Health Policy and Strategies, the exisitence of such policies in Ghana proved insufficient for tangible gains as seen in this study(3, 23). Agblevor et al also reported that Ghana’s Adolesent Healthcare is beset with lack of political will for national implementation drive(3). Which could explain the poor quality of care findings in this study almost ten years after such policies were rolled out in Ghana. Poor utilization of health services was reported in the situational analysis in the Ghana National Adolescent Health Service Policy and Strategy, although the said service was not mentioned specifically (23). Kyilleh et al (13) at West Gonja in Northern Region and Hagan et al (14) in Kumasi, Ashanti region of Ghana both reported low ultilisation of reproductive and sexual health services. In this study, reproductive and sexual health was identified as important health needs of adolescents and young adults, yet none of the AYAs recommended that it should be added to the package of services and it is difficult to extrapolate a reason for it. Otherwise the other findings in this study revealed that AYAs positively gravitate towards high quality clinical care. Hostile adults whose attitude make healthcare services unfriendly to adolescents was also reported by Mbalinda et al in Uganda(21) and Kyilleh et al(13) and Hagan et al(14) in Northern and Ashant regions of Ghana respectively. Just as found in this study, they also reported that repulsive healthcare workers’ behavior creates fear in Adolescents and made these healthcare workers unapproachable. Adolescent place much premium on Automony, Privacy and confidentiality. In a study by Oppong-Odiseng et al(39), AYAs did not trust school clinics (because they were near to where familiar people were found, and also due to close proxity to their familiar environment) as safe enough to provide them adequate privacy and confidentiality. The need for safe environments that provide privacy and confidentiality was seen in other studies to be so crucial in our Ghanaian context for uptake of sexual and reproductive health services (2, 14, 15, 39). Although in this study that was not specifically explored, but the need to provide care that ensures AYAs have adequate privacy and confidentiality, and that which gives them autonomy was evident. This study also shed light on the readiness of both AYAs and Guardans to embrace psychosocial history taking that can help unearth health issues in Adolescents early. This area was not yet explored by studies done in Ghana.

The authors therefore identified four broad areas for pragmatic action for improvement in Adolescent health services in Northern Ghana and the country as a whole: 1. restructuring mainstream services to become more responsive to adolescent healthcare needs. 2. Strategies to improve health literacy on adolescent health concepts and development among AYAs, guardians and community, 3. Establishment and linkage to specialized services for adolescents and 4. Incorporating Adolescent Health into existing curricula for training. The concept is attached as supplement.

Seeing the disparities in the implementation of various services for adolescents, WHO is now advocating for going “beyond single problem thinking” as they noticed that out of 109 countries reviewed that gave attention to adolescent health, three-quarters focused only on sexual and reproductive health. WHO again emphasized in this document that risk reduction interventions for adolescents for various health problems should be tackled jointly because they share common characteristics or have similar underpinnings(1). We are of the view that restructuring mainstream services to become adolescent-friendly and more responsive is more sustainable than stand-alone services due to human resource, infrastructural and financial constraints. The restructuring will include organizing care that provides comfort, and confidentiality; is appropriate and easily accessible at an affordable cost. The views of Patients and the community must also be incorporated to make it culturally appropriate, convenient, acceptable. This study revealed lapses that should be looked out for and addressed in any facility. Private consultation alone with Young Adults 18years and above should be instituted in routine care. Regular In-service training using national and WHO documents and tools to build requisite knowledge, skill and improve communication among healthcare workers taking care of adolescents in primary care is crucial and can address the infractions observed in the study(40). This training from the Self-Determination Theory perspective will equip staff with knowledge and skills to become more confident and tactful in service delivery. It is expected to promote willingness to offer adolescent-responsive services and thus be seen as autonomous support. Also, it will increase their competencies for improved Adolescent-staff relatedness. In this study, the staff requested for regular in-service training and it is expected that it will lead to sustained intrinsic motivation for improved care if done. There is evidence that supports this expectation as human resource management strategies suggest sustained employee motivation for improved outcomes from this perspective(31, 32, 41–44). This study focused more on the clinical aspect of care, but other aspects of healthcare needs that the restructuring should take into consideration and integrate into mainstream care are sexual and reproductive health service units, mental health units, adolescent nutrition and outreach to schools. Annual school health outreaches at school clinics can be used to address their health risks.(45)

Also, efforts must be made to involve parents and community members as they serve as gatekeepers and can also contribute greatly to social behaviour change and implementation of healthcare interventions(1, 16, 22, 37). Neurodevelopmental psychology evidence explains the heightened risky behaviours such as risky driving, binge drinking, substance use, and unsafe sex during adolescence and this evidence must be used to plan interventions outside the hospitals. What will equip AYAs themselves, guardians and community members is a good understanding of how the imbalance in the reward-seeking regions and the cognitive control network regions of the brain of adolescents impact adolescent behaviour and decision-making. It is known that the unique social influence in adolescents is not found in children and adults in empirical developmental studies(46). The social motivation model posits that valuable social goals increase AYA’s social motivation to demonstrate or conform to peer risky behaviour in order to attain them. It is crucial to understand what these social goals are and what they are not to enable less risky or meaningful roles or responsibilities to be planned to replace them where possible. The reward sensitivity model suggests that “arousal leads to altered reward processing, making risk-taking more appealing”(47). The presence of peers brings this arousal and signalling for rewards and interventions to model good behaviour and decision-making should among others include restricting AYAs from risky gathering or social or media programmes. The distraction model reveals the need for training in mindfulness and meditation to develop desirable behaviour as these have been found to be helpful in reducing and improving depression symptoms among AYAs(48) Youth clubs are ideal avenues for modeling good behavior and can employ role plays to promote risk aversion and meaningful role interventions. Also teachers, social medial, mass media, cell phones all impact adolescents’ lives and health literacy strategies ought to strongly consider using these avenues to reach adolescents and young adults. Peer Education using expert patient education models is a strong incentive for peers to influence other AYAs positively. The current Ghanaian health system lacks structures and programmes that leverages these opportunities for adolescent health literacy and this must be looked at.

The other gap in service delivery is in the area of healthcare transition. Adolescents with chronic diseases such as Type I diabetes, Asthma, Sickle Cell disease, Seizure disorder, and HIV/AIDS usually get lost to care after Paediatric care. Paediatrics and Child health services typically end at age 12 in Ghana. Although Adolescents with these chronic diseases are usually seen by Paediatrics until 18 years old, National Health Insurance reimbursement becomes an issue and the lack of a structured transition plan makes their health outcomes decline afterwards. Staff at adult clinics are typically unfriendly to adolescents(21). It is therefore imperative to have a well-structured transition of care from Paediatric Chronic care clinics to adolescent-oriented or specialist clinics either manned by Physicians with training in adolescent healthcare or adolescent medicine physician specialists. The integration of services talked about earlier must also occur in these clinics where all their needs are collaboratively addressed.

The current late training approach whereby Adolescent Medicine which gives much attention to adolescent healthcare is reserved for Fellowship training arguably contributes greatly to why the existing healthcare system serves adolescents least well(49, 50). As one of the participants aptly put it above; a young Population like Ghana’s should position its healthcare system properly to take good care of its future workforce which constitutes more than half of its population. This view point is strongly endorsed by WHO(40) and Kokotailo et al(50). Unfortunately, this is not the case in Ghana and many African countries. It is expedient that pre-service training for all healthcare workers should be rebost enough in adolescent health to make healthcare workers competent and skillful. Although specialized care clinicians are needed for complex stand-alone practice settings, in our view what best suits our healthcare system is a better pre-service training in adolescent health. Therefore we propose a model for training and integration of services where every qualified healthcare worker gains the requisite knowledge, skill and attitude for quality adolescent healthcare services. Postgraduate is necessary just as in other disciplines but should not be the only form of training, often awaited, and in many places unavailable.

## STRENGTHS AND LIMITATIONS

The procedure employed to recruit and interview participants and analyze the data ensured across service points inclusion and all clinical service aspects assessment both from patients, guardains and staff members. Rigor and quality measures were employed in designing interview guide, choosing analytic direction and analysis and manuscript write up. The limitations of the study are potential social desirability bias and inability to give participants opportunity to validate the themes generated.

## CONCLUSION

AYA and guardians want convenient, easily accessible and high-quality care, delivered by a friendly and trusted team; which views the healthcare workers also endorsed. To improve adolescent health services in Ghana, healthcare facilities and training institutions should restructure their healthcare and curricula along global quality standards.

## Supplementary Material

Supplementary Files

This is a list of supplementary files associated with this preprint. Click to download.

• ADOLESCENTSERVICEDELIVERYPATHWAYschema.pdf

• FinalInterviewGuideforKIandfocusGroups.docx

## Figures and Tables

**Table 1:** Demographic data

Demographic data (N = 42)	AYA	Guardians	Healthcare staff	Total
**Age Range (Frequency)**	14–24 years (12)	32–64years (8)	28–55years (22)	(42)
**Sex**				
Male	(4)	(3)	(14)	(21)
Female	(8)	(5)	(8)	(21)
**Level of Education**				
JHS	(1)	(3)		(4)
SHS	(5)	(1)		(6)
Tertiary	(6)	(4)	(22)	(32)
**Occupation**				
Unemployed	(11)	(4)		(15)
Employed	(1)	(4)	(22)	(27)
**Type of Interaction**				
FGD	No	No	Yes	(22)
In-depth interview	Yes	Yes	No	(20)

## Data Availability

The datasets and materials used and/or analysed during the current study are available from the corresponding author on reasonable request.

## Abbreviations

NYU: New York University
NY: New York
U.S: United States
USA: United States of America
AYA: Adolescent and Young Adult
WHO: World Health Organisation
OPD: Outpatient Department
FGD: Focused Group Discussion
LHIMS: Lightwave Health Information Management System
HEEADSSS: Home environment, Education and Employment, Eating and Exercise, Peer-related Activities, Drugs, alcohol, tobacco, Sex and Sexuality, Suicide, depression and other mental related issues and Safety from injury, violence, abuse
HIV/AIDS: Human Immunodeficiency Virus/Acquired Immunodeficiency Syndrome
